# Comparative Examination of the Olive Mill Wastewater Biodegradation Process by Various Wood-Rot Macrofungi

**DOI:** 10.1155/2014/482937

**Published:** 2014-06-02

**Authors:** Georgios Koutrotsios, Georgios I. Zervakis

**Affiliations:** Laboratory of General and Agricultural Microbiology, Agricultural University of Athens, Iera Odos 75, 11855 Athens, Greece

## Abstract

Olive mill wastewater (OMW) constitutes a major cause of environmental pollution in olive-oil producing regions. Sixty wood-rot macrofungi assigned in 43 species were evaluated for their efficacy to colonize solidified OMW media at initially established optimal growth temperatures. Subsequently eight strains of the following species were qualified: *Abortiporus biennis*, *Ganoderma carnosum*, *Hapalopilus croceus*, *Hericium erinaceus*, *Irpex lacteus*, *Phanerochaete chrysosporium*, *Pleurotus djamor*, and *P. pulmonarius*. Fungal growth in OMW (25%v/v in water) resulted in marked reduction of total phenolic content, which was significantly correlated with the effluent's decolorization. *A. biennis* was the best performing strain (it decreased phenolics by 92% and color by 64%) followed by *P. djamor* and *I. lacteus*. Increase of plant seeds germination was less pronounced evidencing that phenolics are only partly responsible for OMW's phytotoxicity. Laccase production was highly correlated with all three biodegradation parameters for *H. croceus*, *Ph. chrysosporium*, and *Pleurotus* spp., and so were manganese-independent and manganese dependent peroxidases for *A. biennis* and *I. lacteus*. Monitoring of enzymes with respect to biomass production indicated that *Pleurotus* spp., *H. croceus*, and *Ph. chrysosporium* shared common patterns for all three activities. Moreover, generation of enzymes at the early biodegradation stages enhanced the efficiency of OMW treatment.

## 1. Introduction

Three-phase centrifugal olive mills are widespread in most olive-oil producing countries and their operation results in the production of olive oil, solid pomace, and olive mill wastewater (OMW). The disposal of the latter creates major environmental problems since it is rich in organic compounds and contains large amounts of polyphenolics in high (and therefore) toxic concentrations [1, 2]. In addition, the olive mills seasonal operation, small size, and scattered distribution hinder the effective management of the effluent [3].

Among the large variety of methodologies adopted for the treatment of OMW [4–7], implementation of biological approaches is of particular importance. For example, the use of microorganisms could result in both the detoxification of the effluent and the generation of value-added products, for example, xanthan gum, citric acid, ethanol, antioxidants, polyhydroxyalkanoates, b-glucan, lipase, single cell oil, and edible biomass [8–18].

In particular, wood-rot macrofungi constitute a group of organisms equipped with a potent biochemical arsenal including one or more nonspecific groups of enzymes, which permits them to effectively decompose various lignocellulosic compounds [19–21]. The chemical affinity of the latter to a wide range of agroindustrial wastes (including OMW) led to their treatment with filamentous basidiomycetes originally isolated from woody substrates. Therefore, such organisms were examined either at a OMW pretreatment stage for decreasing the effluent's high content of phenolics and hence toxicity prior to its processing with other microorganisms [22, 23] or as the main/sole agents for OMW's remediation [24–27]. However, most pertinent investigations have focused on the exploitation of a few species only, for example,* Phanerochaete chrysosporium*,* Ph. flavido-alba*,* Pleurotus ostreatus,* and* P. eryngii* [28–32], and consequently a large number of other potentially useful macrofungi remain understudied or not examined at all.

The objective of the present work was the comparative evaluation of a wide variety of mushroom fungi for OMW biotreatment and the subsequent establishment of a battery of strains able to efficiently degrade OMW. The process was assessed by determining the activities of ligninolytic enzymes produced by qualified organisms at different time periods and through their association with concomitant OMW decolorization, reduction of phenolics, and decrease of plant toxicity.

## 2. Materials and Methods

### 2.1. Fungal Strains

Sixty wood-rot macrofungi belonging to 43 species of basidiomycetes (phylum Basidiomycota) were evaluated for the purposes of this study. Details of the strains identity are presented in Table 1. All the biological material is maintained in the fungal culture collection of the authors institution (AUA-LGAM).

### 2.2. Nutrient Media: Fungal Growth Substrates and Conditions

OMW was obtained from an olive-oil mill equipped with three-phase centrifugal decanters located in Kalamata (Peloponnese, S.W. Greece). Its composition and main physicochemical properties were previously assessed [2].

For the preparation of OMW-containing growth media, the effluent was adjusted at pH 6 by adding CaO and centrifuged for 20 min at 5000 g (5°C), and the supernatant was diluted or not with deionized water to prepare 25%, 50%, and 100% v/v OMW-based substrates, which were further heat-sterilized for 30 min (121°C, 1.1 atm). Fungal strains were subcultured on a 25% v/v OMW medium solidified with 1.7% w/v agar. Then, agar plugs (6 mm diam.) originating from the actively growing part of the fungal colonies were used to inoculate either solidified OMW media (for the purposes of mycelium growth rate determinations) or static liquid cultures (for OMW degradation experiments with selected strains).

### 2.3. Screening of Fungal Strains: Establishment of Temperature Optima and Measurements of Linear Growth Rates

The temperature optima for mycelium linear growth were assessed on potato dextrose agar (PDA; Conda) medium and over the range of 12–47°C with a 5°C graduation. Experiments were conducted in Petri dishes as previously described [33], and measurements were taken every 24 h for all species examined with the exception of* Phanerochaete chrysosporium* and* Hapalopilus croceus* strains whose growth was measured every 12 h until seven consecutive values were obtained for all strains.

Growth rates were determined by measuring the distance of the colony's front from the centre of the inoculum at four different points along two perpendicular lines [34]. Mycelium growth rates (*k*_*r*_) were calculated by fitting the linear growth function *y* = *k*_*r*_ *x* + *c* (where *y* is the distance covered by the hyphae and *x* is the respective time) and were expressed in mm d^−1^. All experiments were conducted in three replicates.

After establishing the optimal temperature for mycelium growth for each strain, all cultures were evaluated with respect to their growth rates on various OMW dilutions (25%, 50%, and 100% v/v OMW in water) at their optimal temperatures as previously determined, in the same way as described above. In addition, PDA was used as control. All mycelium growth rate experiments were conducted in three replicates.

### 2.4. Treatment of OMW by Qualified Fungal Strains: Assessment of Effluent's Selected Properties

The strains, which were qualified from the previous experiment, were further examined as regards their effect on OMW degradation process in liquid static cultures. The latter were performed in 250 mL Erlenmayer flasks containing 100 mL of 25% v/v OMW, at the optimal temperatures previously established for each strain, and lasted for a period from 20 to 30 days depending on the growth of each fungus, that is,* Abortiporus biennis* ABL436 (25 days),* Ganoderma carnosum* GCL448 (25 days),* Hapalopilus croceus* HCC522 (20 days),* Hericium erinaceus* HEL801 (25 days),* Irpex lacteus* ILC238 (25 days),* Phanerochaete chrysosporium* PHL322 (20 days),* Pleurotus djamor* PDC855 (30 days), and* P. pulmonarius* PPL111 (30 days). Four replicates were included for each treatment, while noninoculated substrates were also incubated in parallel and served as controls.

A destructive sampling process was adopted at five time points for dividing the entire cultivation at intervals of four, five, or six days (for growth periods of 20, 25, or 30 days, resp.), in order to determine pH, electric conductivity, biomass, total phenolics, decolorization, plant seed germination, and enzymes activity.

Measurements of pH and electric conductivity in OMW were performed by using a Corning EEL 12 pH meter and a Jenway 4010 conductivity meter, respectively. Biomass was harvested by filtration and its weight (mycelium dry weight) was determined by drying at 60°C until constant weight.

Total phenolics were analyzed by the Folin-Ciocalteu method [35], by measuring spectrophotometrically (at 760 nm) the formation of a blue complex as a result of the reduction of a phosphomolybdic-phosphotungstic reagent from the presence of phenolics. The concentration of total phenolics was determined against a syringic acid calibration curve (1 mg mL^−1^ syringic acid—initial concentration—resulted in an optical density of 0.377 at 760 nm).

Decolorization was estimated by measuring the absorbance of OMW samples at 525 nm using a U-2001 spectrophotometer (Hitachi Instruments Inc., USA).

The plant seed germination indexes were estimated on the basis of Zucconi et al. [36] protocol. Twenty-five cress seeds (*Lepidium sativum* L.) were placed on filter papers moistened with OMW (25%) and incubated in Petri dishes for three days at 25°C. The effect of OMW on germination was evaluated, and germination indexes (G.I.) were calculated as follows: G.I. = (% root elongation × % germination)/100 (control used: cress seeds moistened with water).

### 2.5. Determination of Enzymes Activities

Laccase (Lac, E.C. 1.10.3.2: benzenediol: oxygen oxidoreductase) activity was determined at 425 nm by oxidizing 0.4 mL ABTS (2,2-azino-bis(3-ethylbenzothiazoline-6-sulphonic acid)) (1.5 mM) with 0.8 mL of OMW sample in 1.2 mL Na-tartrate buffer (0.1 M, pH 4.5) [37].

Manganese-independent peroxidase (MnIP) was measured at 590 nm by the oxidative coupling of 0.1 mL MBTH (3-methyl-2-benzothiazoline hydrazone) (1 mM) and 0.2 mL DMAB (3-dimethylaminobenzoic acid) (25 mM) in the presence of 0.01 mL H_2_O_2_ (10 mM) added to a solution consisting of 0.66 mL sample and 1 mL succinate-lactate buffer (0.1 M, pH 4.5), while background activity, determined as above in the absence of H_2_O_2_, was subtracted [32].

Manganese peroxidase (MnP, E.C. 1.11.1.13 (MnII): hydrogen peroxide oxidoreductase) activity was determined as described for manganese-independent peroxidase (MnIP) in the presence of 0.01 mL MnSO_4_ (20 mM), by subtracting MnIP activity [38]. For all enzymes determination, one activity unit was defined as the amount of enzyme transforming 1 *μ*mol of substrate per minute.

### 2.6. Statistical Analysis

Analysis of variance (ANOVA) followed by Gabriel's multiple comparison tests (*a* < 0.05) was used to estimate statistical differences between treatment means through the use of SPSS (version 18) software. Standard deviations were calculated for all mean values, and regression analysis was carried out to evaluate relationships between variables at significance levels of 1% and 5%.

## 3. Results and Discussion

**Figure 1:**
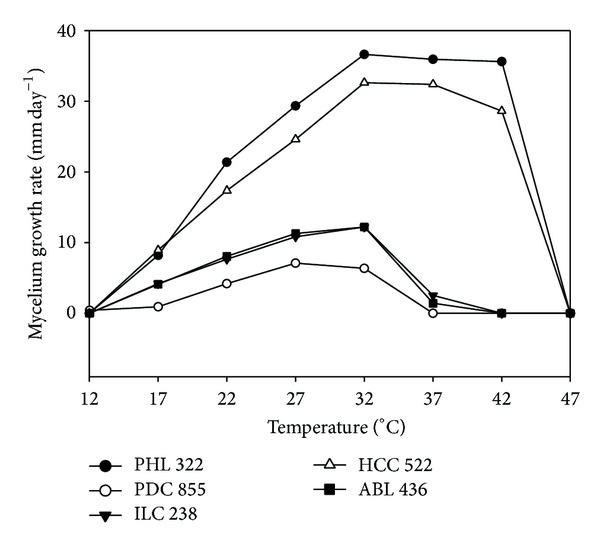
Mycelium linear growth rates (*K*_*r*_, mm day^−1^) on PDA as measured at different temperatures (°C) for selected indicative cases of macrofungi examined (i.e.,* A. biennis* ABL436,* H. croceus* HCC522,* I. lacteus* ILC238,* Ph. chrysosporium* PHL322, and* P. djamor* PDC855).

Data about the optimal conditions for the production of fungal biomass are available for a rather few species of macrofungi (i.e., mainly those exploited for edible mushroom production). The effect of temperature, in particular, is of fundamental importance in evaluating the biotechnological potential of such organisms [33, 34], and hence it was the first parameter which was assessed in the present study. The results of the comparative mycelium linear growth rate examination covering a wide temperature range (12–47°C) showed that most strains presented their optima at a narrow value area of 27–32°C (Table 2). Only* Hypsizygus ulmarius*,* Omphalotus illudens*,* Pholiota nameko,* and* Pleurotus nebrodensis* exhibited significantly higher growth rates at 22°C. On the other hand,* Phanerochaete chrysosporium* PHC571 was the only one presenting optimal growth at 37°C. It is noteworthy that almost all* Pleurotus* strains showed a growth (albeit limited) at 12°C;* Pleurotus* was the only genus among the 28 different genera evaluated demonstrating such a property. In contrast,* Hapalopilus croceus* and* Phanerochaete chrysosporium* were the only species showing hyphal development at the other temperature extreme (42°C). Furthermore, mycelium growth rates of almost all strains presented a gradual increase from lower temperatures to the optimal one and then a sharp decrease to the temperature of no growth; in fact, this effect was more pronounced for individuals demonstrating high growth values (Figure 1).

In the next comparative evaluation test, the mycelium growth rates of all strains were measured at their optimal temperatures and on three different OMW concentrations (25%, 50%, and 100% OMW v/v, in water) plus on PDA (Table 3). As anticipated, mycelium growth on PDA was in all cases significantly higher than on OMW-based solidified media with the only exception of* Ganoderma carnosum* GCL448 growing on 25% OMW. Still, several other strains presented satisfactory growth on both 25% and 50% OMW, while some performed adequately on 100% OMW as well. Such noteworthy cases included (apart from GCL448)* Abortiporus biennis*,* Coriolopsis trogii, Daedalea quercina, Ganoderma adspersum, G. resinaceum* (GRL344 and GRL403),* Hapalopilus croceus, Hericium erinaceus* (HEL801 only),* Irpex lacteus, Phanerochaete chrysosporium, Pleurotus djamor, P. pulmonarius, Stereum hirsutum, Trametes versicolor,* and* Tyromyces lacteus*. On the other hand, practically no growth was detected on OMW-based media for* Auricularia mesenterica*,* Fistulina hepatica*,* Grifola frondosa*,* Hericium erinaceus* (HEL802 only),* Hypsizygus ulmarius*,* Laetiporus sulphureus* (LSL331 only),* Lentinula edodes*,* Pholiota nameko,* and* Pleurotus abieticola.* In some of the rather few cases in which a species was represented by more than one individual, results occasionally revealed much different behavior between certain conspecific strains, for example, in* Ganoderma carnosum*,* Hericium erinaceus,* and* Laetiporus sulphureus*. This observation is in accordance with previous findings reporting high intraspecific variability within* Pleurotus* spp. in the degradation of OMW phenolics [32], which advocates for a strain (rather than a species) dependent behavior.

Consequently, eight fungi (i.e.,* Abortiporus biennis* ABL436,* Ganoderma carnosum* GCL448,* Hapalopilus croceus* HCC522,* Hericium erinaceus* HEL801,* Irpex lacteus* ILC238,* Phanerochaete chrysosporium* PHL322,* Pleurotus djamor* PDC855, and* P. pulmonarius* PPL111) were qualified for further study on the basis of the outcome of the previous experiment, on the effect their growth had on the decolorization of the OMW-based substrate (assessed by visual inspection, data not shown), and on the prospects that individual species present in their subsequent/future exploitation for the generation of value-added products (e.g., edible biomass from* Pleurotus* spp.).

In all cases, fungal growth resulted in pH decrease from an initial value of 5.65 to values of as low as 4.48 for* I. lacteus*, while for the rest of the strains it ranged from 5.00 to 5.50 (Table 4). However, differences in pH were less pronounced for* Pleurotus* spp., which is in accordance with previous pertinent results obtained after the use of* P. ostreatus* [39]. On the other hand, electric conductivity measurements demonstrated a gradual increase from 1.80 mS cm^−1^ in the initial material to 3.85–5.97 mS cm^−1^ in the biodegraded effluent (Table 4); this seems to be the general trend for conductivity values after microbial treatment of raw OMW [40].

**Figure 2:**
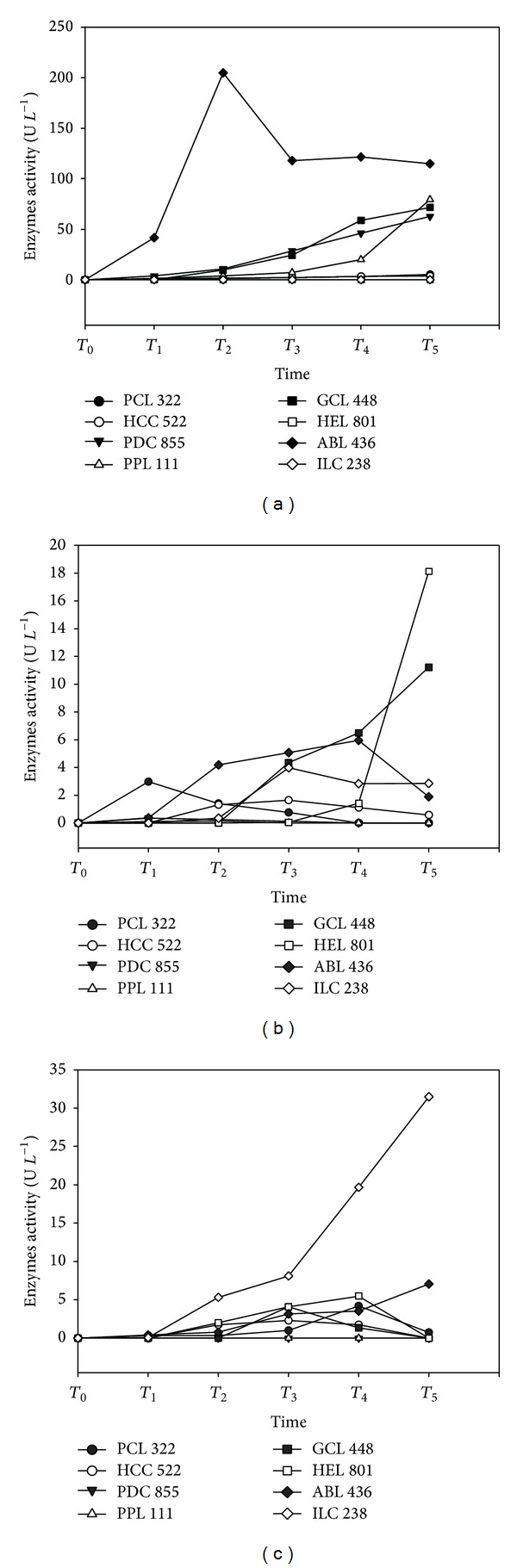
Enzyme activities (U L^−1^) for laccase (a), manganese peroxidase, (b) and manganese-independent peroxidase (c) exhibited on OMW-based media (25% v/v in water) by eight selected macrofungi during a growth period of 20 to 30 days and in five different time points (*T*_1_ to *T*_5_) as follows: 4, 8, 12, 16, and 20 days for* H. croceus* HCC522 and* Ph. chrysosporium* PHL322; 5, 10, 15, 20, and 25 days for* A. biennis* ABL436,* G. carnosum* GCL448,* H. erinaceus* HEL801, and* I. lacteus* ILC238; 6, 12, 18, 24, and 30 days for* P. djamor* PDC855 and* P. pulmonarius* PPL111.

All selected strains produced abundant mycelium in static batch cultures; yet four out of the eight strains (*A. biennis, H. croceus, P. djamor,* and* P. pulmonarius*) provided significantly higher yields (200–230 mg) than the rest (Table 4). Nevertheless, biomass production at initial growth stages was rather poor since substrate's content in readily assimilated nutrients was low [2]. In general, fungal growth was accompanied with varying degrees of enzymes production among the various strains examined.* A. biennis* was the only fungus to demonstrate a particularly high laccase activity within the initial cultivation period; that is, a peak value of 205 U L^−1^ was obtained after 10 days only, and then it subsided and remained rather constant at values of 115–122 U L^−1^ until the end of the cultivation (Figure 2(a)). The rest of the strains demonstrated either significantly lower laccase activities or no activities at all. Within the former group, laccase production for both* Pleurotus* spp. and* G. carnosum* increased in parallel with biomass growth and reached the highest value by the end of cultivation (63–79 U L^−1^), whereas* H. croceus* and* Ph. chrysosporium* presented much lower values (3.6–5.1 U L^−1^). In contrast, in the cases of* H. erinaceus* and* I. lacteus* no laccase activity was detected.

As regards Mn peroxidase (MnP), a large variability was detected among strains in terms of both the peak activity values and the time periods where these were produced (Figure 2(b)). Hence, the highest MnP activities were observed for* H. erinaceus* and* G. carnosum* (18 and 11 U L^−1^, resp.) at the end of their growth.* A. biennis*,* H. croceus,* and* I. lacteus* presented their peak MnP values at or near the middle of the cultivation period (1.6–5.9 U L^−1^). On the other hand,* Ph. chrysosporium* and the two* Pleurotus* spp. were early MnP producers since their maximum production (0.4–3.0 U L^−1^) was noted within the first 12 days of growth, and then it gradually subsided to levels of no detection at the end of cultivation. Production of MnP was previously associated with secondary metabolism activated by lack of adequate nitrogen and/or carbon [41]. This was apparently not the case during the early stages of incubation of* Ph. chrysosporium* and* Pleurotus* spp. used in this study, but it might well be the case for the other five strains which presented their peak activities with a time delay. On the other hand, no Mn independent peroxidase (MnIP) activities were detected by the two* Pleurotus* spp. tested (Figure 2(c)). All the other strains produced their peak MnIP values towards the second half of their growth period starting from day 12 (*H. croceus*, 2.3 U L^−1^) and ending at day 25 (*Irpex lacteus* and* A. biennis*, 32 U L^−1^ and 7.1 U L^−1^, resp.).

In general, monitoring of enzymes production with respect to fungal growth indicated that* P. djamor* and* P. pulmonarius* shared identical patterns for all three activities, and this was also the case for* H. croceus* and* Ph. chrysosporium.* On the other hand,* A. biennis* presented common production patterns with* I. lacteus* as regards MnP and MnIP and so did* G. carnosum* with* H. erinaceus.*

Although in some cases the findings of the present study are in line with previous literature reports on enzyme activities in relation to fungal growth, for example, MnP activity for* Pleurotus* and* Ganoderma* spp. [32, 39], different types of data were also obtained. For example, MnIP activities in* Ganoderma* spp. showed their peak production earlier during the growth period [32] in comparison to the findings of the present study. Furthermore, previous pertinent results on* P. eryngii* and* P. ostreatus* revealed late production of MnP [32], which is in contrast with the results of this work (albeit with different* Pleurotus* spp.). While differences in enzyme activity values among strains used in various investigations could be attributed to both experimental conditions (including initial substrate composition and dilution rate) and properties of the particular fungus used, variations in enzyme production with respect to time could be primarily explained by the latter factor (i.e., variability of the biological material employed).

Total phenolic content was markedly reduced by all strains; however, particularly impressive was the reduction achieved by* A. biennis*, which after only six days of incubation decreased the initial phenols by more than 50%, while by the end of the experiment the respective figure reached 92% (Table 4). Very good performers (although significantly inferior than the first) were the two* Pleurotus* species (71%–76% of decrease), followed by* I. lacteus* and* G. carnosum*. The latter was the second fastest (after* A. biennis*) in reducing total phenols since a 49% decrease was measured after just 12 days of incubation. It is noteworthy that other* Ganoderma* species/strains were reported to present similar behavior as regards the time needed to decrease to a similar extent OMW's phenolics, that is, by 41–44% within a 10-day incubation period and by reaching a total reduction exceeding 64% [26, 32]. On the other hand, selected* P. ostreatus* and* P. eryngii* strains demonstrated a rather delayed phenols degradation which exceeded the 50% level only after the completion of the first half of growth [32] much like as it was observed for* P. djamor* and* P. pulmonarius* in the present study. In certain cases (e.g., for* A. biennis*,* G. carnosum,* and* I. lacteus*), a notable deceleration in the reduction rate of total phenols reduction was observed during the second half of the growth period, which could be attributed to the recalcitrance of certain phenolics remaining in culture (e.g.,* trans*-cinnamic acids which are not degraded by laccase) [42].

Of special interest was that total phenol reduction was significantly correlated with laccase and MnP activities (*r* ^2^ = 0.43 and *r* ^2^ = 0.71, resp.) when data from all eight fungi were collectively evaluated (Table 5), while these figures were found to be particularly high in the individual calculations made for* H. croceus*,* Ph. chrysosporium,* and* Pleurotus* spp. for laccase (*r* ^2^ = 0.94–1.00),* A. biennis*,* G. carnosum*,* H. erinaceus,* and* I. lacteus* for MnP (*r* ^2^ = 0.71–0.94), and* A. biennis*,* I. lacteus,* and* Ph. chrysosporium* for MnIP (*r* ^2^ = 0.81–1.00). Such correlations were detected not only during the biodegradation of OMW by* P. ostreatus* [32, 39] but also when effluents from the debittering of green olives were treated by several white-rot fungi [43]. In this category of microorganisms, production of laccase is often induced by the presence of the appropriate substrate in the growth environment [44]. Especially as regards OMW media, such induction for* Pleurotus* spp. was associated with the concentration of the effluent in the culture substrate; high laccase activities were detected when phenolics exceeded 1.5 g L^−1^ [39, 45, 46], which is also the case with the initial phenolics content in the samples of this study (>2.0 g L^−1^). Most of the fungi examined here (with the only exception of* A. biennis*) demonstrated a lag phase for laccase production indicative of the time they needed to adapt their growth requirements to OMW [24, 47].

As previously noted with phenols content,* A. biennis* was the most efficient fungus in decolorizing OMW by reaching a 64% value by the end of the cultivation period (Table 4). During the process, color increased (for this and some other strains) during the first measurement(s) as a consequence of the oxidation and/or polycondensation of phenolic compounds in other darker-colored forms [44, 48]. Still,* A. biennis* achieved fast a very high decolorization rate (48% after 12 days) which is significantly higher than the respective values obtained from the other fungi during that same period (2%–18%).* I. lacteus* and* Pleurotus* spp. provided the next best total decolorization (56% to 39%), whereas* G. carnosum* performed rather poorly (14%). On the other hand,* H. erinaceus* was the only fungus that performed significantly better during the first half of the growth period, and then its decolorization subsided considerably.

OMW decolorization efficacy by* A. biennis* is among the best reported in literature for basidiomycetes. In other pertinent studies,* Pleurotus* spp.,* Ganoderma applanatum*,* Lentinula edodes, Pycnoporus coccineus, Coriolopsis polyzona,* and* Lentinus tigrinus* demonstrated similar magnitude of decolorization; however, in several of these cases initial medium and cultivation conditions varied considerably [26, 32, 49, 50]. In addition, the outcome of this study demonstrates that decolorization is significantly correlated with total phenol reduction (*r* ^2^ = 0.71), which is in line with previous results associating OMW decolorization with the degradation of high molecular-mass polyphenols [47, 51]. Furthermore, high correlations were detected for decolorization not only versus laccase activity for majority of the fungi examined (*r* ^2^ = 0.83–0.99, in the cases of* G. carnosum*,* H. croceus*,* Ph. chrysosporium,* and* Pleurotus* spp.), but also versus MnP (*r* ^2^ = 0.66–0.99, for* A. biennis*,* G. carnosum*,* H. erinaceus,* and* I. lacteus*) and versus MnIP (*r* ^2^ = 0.83–0.99, in the cases of* G. carnosum*,* I. lacteus,* and* Ph. chrysosporium*). In the past, OMW decolorization was associated with laccase production for other white-rot fungi as well [24, 32, 52].

As regards the phytotoxicity evaluation,* A. biennis* was again the best performer by demonstrating 30% increase of the germination index with respect to untreated OMW, while* G. carnosum*,* I. lacteus,* and* H. erinaceus* provided significantly lower values (15%–18%). In these cases, increase of plant seed germination was particularly slow during the first 1 to 2 weeks of incubation, which is in line with previous reports on* P. ostreatus* and* P. eryngii* achieving 19%–27% increase of germination index towards the end of the growth period on OMW [32]. On the other hand, the other four strains produced a very low increase in seed germinability (<10%) that could be possibly attributed to the formation of phenoxy radicals and/or quinonoids that are more toxic than the initial phenolic compounds [53, 54]. In general, toxicity of OMW was associated with the presence of aromatic compounds of low molecular weight and with synergistic inhibition caused by phenolics [55–57]. Increase in seed germination was significantly correlated with reduction in phenolics and decolorization (*r* ^2^ = 0.84 and 0.71, resp., in cumulative calculations made for all strains tested); this high correlation with the former factor is in accordance with past reports on the germination of* Triticum* and* Lepidium* plant seeds in OMW treated by* L. edodes* and* Pleurotus* spp., respectively [24, 32].

When the results presented above were reassessed by taking into consideration the time needed for obtaining the final values for total phenols reduction, decolorization, and increase of plant seed germination, classification of selected strains did not change considerably. For example,* A. biennis* was again significantly more efficient for all parameters evaluated; that is, it demonstrated a daily average of 3.70 percentage units of total phenols reduction, 2.56 percentage units of decolorization, and 1.20 percentage units of seed germination increase;* Pleurotus* spp.,* G. carnosum*,* H. croceus,* and* I. lacteus* followed as regards total phenols reduction (with values of 2.35–2.53),* I. lacteus* for decolorization (2.24), and* G. carnosum*,* I. lacteus,* and* H. erinaceus* for seed germination increase (0.60–0.70).

**Figure 3:**
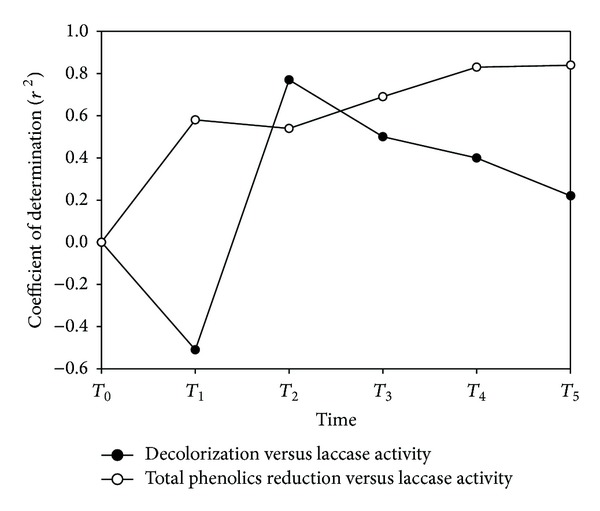
Coefficient of determination (*r* ^2^) values for OMW decolorization and total phenolics reduction versus the laccase activities presented by the eight selected macrofungi as calculated from the respective data obtained at five time points (*T*_1_ to *T*_5_) during their entire growth period.

It is particularly noteworthy that when the experimental data from all eight fungi were assembled and evaluated vis-à-vis their total phenol reduction, decolorization, plant seed germination increase, and enzymes activity (Table 5), then biomass and germination index showed statistically significant correlations in all comparisons (*r* ^2^ = 0.55–0.88 and *r* ^2^ = 0.51–0.88, resp.). Laccase production was significantly correlated with total phenolics reduction and decolorization (Figure 3), and so was decolorization with MnIP (*r* ^2^ = 0.46). Last, MnP, and MnIP activities were also correlated (*r* ^2^ = 0.55).

## 4. Conclusions

All wood-rot macrofungi selected for the purposes of this study were particularly effective in colonizing and degrading OMW through an efficient enzyme-producing mechanism. Especially* A. biennis* and secondly* I. lacteus*,* G. carnosum,* and* Pleurotus* spp. achieved total phenol reduction and decolorization values, which were significantly correlated with laccase and/or peroxidases activities. Enhancement of plant seed germination was less pronounced but it demonstrated high correlation with the other two biodegradation parameters. This approach seems to be suitable (alone or in combination with other techniques) in the development of a sustainable methodology leading to the efficient treatment of OMW.

## Figures and Tables

**Table 1:** Details of the biological material evaluated for the purposes of this study.

Species	Geographic origin and host/substrate	Collection code
*Abortiporus biennis *	Czech Republic	ABC521
*Abortiporus biennis *	Greece	ABL436
*Agrocybe cylindracea *	China	ACL834
*Auricularia mesenterica *	Greece; *Cupressus* sp.	AML472
*Coriolopsis trogii *	Greece; *Quercus* sp.	CTL447
*Daedalea quercina *	Czech Republic	DQC528
*Dichomitus squalens *	Czech Republic	DSC750
*Fistulina hepatica *	Greece; *Castanea sativa *	FHL295
*Flammulina velutipes *	Commercial strain	FVS803
*Fomitopsis pinicola *	Greece; *Abies cephalonica *	FPL302
*Ganoderma adspersum *	Greece; *Abies cephalonica *	GAL401
*Ganoderma carnosum *	Greece	GCL642
*Ganoderma carnosum *	Greece; *Olea sativa *	GCL448
*Ganoderma pfeifferi *	Greece; *Fagus sylvatica *	GPL336
*Ganoderma resinaceum *	Greece; *Morus alba *	GRL334
*Ganoderma resinaceum *	Greece; *Salix babylonica *	GRL403
*Ganoderma resinaceum *	Czech Republic	GRC604
*Grifola frondosa *	Commercial strain	GFS805
*Hapalopilus croceus *	Czech Republic	HCC522
*Hericium erinaceus *	Greece	HEL801
*Hericium erinaceus *	Greece;* Quercus pubescens *	HEL802
*Heterobasidion annosum *	Greece; *Abies cephalonica *	HAL340
*Hypsizygus ulmarius *	Greece; *Abies cephalonica *	HUL417
*Inocutis tamaricis *	Greece; *Tamarix hampeana *	ITL314
*Irpex lacteus *	Czech Republic	ILC238
*Laetiporus sulphureus *	Greece; *Castanea sativa *	LSL331
*Laetiporus sulphureus *	Greece; *Castanea sativa *	LSL332
*Lentinula edodes *	Commercial strain	LES812
*Neolentinus lepideus *	Greece; *Pinus nigra *	NLL317
*Omphalotus illudens *	Greece; *Quercus *sp.	OIL347
*Perenniporia fraxinea *	Greece; *Populus alba *	PFL346
*Phanerochaete chrysosporium *	Greece	PHL322
*Phanerochaete chrysosporium *	Czech Republic	PHC571
*Pholiota nameko *	commercial strain	PNS806
*Pleurotus abieticola *	Russia	PAC854
*Pleurotus citrinopileatus *	Malaysia	PCC884
*Pleurotus cornucopiae *	Iran	PCL660
*Pleurotus cystidiosus *	USA;* Populus deltoides *	PCC897
*Pleurotus cystidiosus *subsp.* abalonus *	China	PAC891
*Pleurotus djamor *	Malaysia	PDC855
*Pleurotus eryngii *	Czech Republic	PEC847
*Pleurotus eryngii *var.* eryngii *	Greece; *Eryngium *sp.	PEL063
*Pleurotus eryngii *var.* eryngii *	Italy*; Eryngium campestre *	PEC810
*Pleurotus eryngii *subsp.* tuoliensis *	China	PEC856
*Pleurotus flabellatus *	Malaysia	PFC860
*Pleurotus nebrodensis *	Greece; *Prangos ferulacea *	PNL126
*Pleurotus nebrodensis *	Italy; *Prangos ferulacea *	PNC816
*Pleurotus ostreatus *	Italy	POC847
*Pleurotus ostreatus *	Czech Republic	POC843
*Pleurotus ostreatus *	Greece; *Abies cephalonica *	POL067
*Pleurotus pulmonarius *	Greece; *Fagus sylvatica *	PPL111
*Pleurotus pulmonarius *	France	PPC823
*Pleurotus pulmonarius *	Hong Kong	PSC757
*Pleurotus tuber-regium *	Papua New Guinea	PTC822
*Stereum hirsutum *	Czech Republic	SHC608
*Trametes hirsuta *	Czech Republic	THC610
*Trametes ljubarskyi *	Greece; *Populus* sp.	TLL473
*Trametes versicolor *	Greece	TVL469
*Trametes versicolor *	Czech Republic	TVC614
*Tyromyces lacteus *	Czech Republic	TLC616

**Table 2:** Mycelium linear growth rates (*K*_*r*_, mm day^−1^) for 60 strains of macrofungi established at different temperatures ranging from 12 to 47°C (ngd: no growth detected). Values are expressed as means ± standard deviation of means, *n* = 3. Lack of superscript letters commonly indicates statistically significant differences (Gabriel's *t*-test, *P* < 0.05) for comparisons of treatment means between different temperatures for each strain.

Strain	Temperature							
	12	17	22	27	32	37	42	47
ABC521	ngd	4.06 ± 0.04^d^	7.46 ± 0.07^c^	11.47 ± 0.07^a^	10.17 ± 0.07^b^	1.47 ± 0.07^e^	ngd	ngd
ABL436	ngd	4.10 ± 0.18^d^	8.03 ± 0.03^c^	11.30 ± 0.08^b^	12.19 ± 0.29^a^	1.39 ± 0.08^e^	ngd	ngd
ACL834	ngd	1.64 ± 0.04^c^	3.12 ± 0.13^b^	4.02 ± 0.10^a^	3.06 ± 0.08^b^	ngd	ngd	ngd
AML472	ngd	ngd	6.24 ± 0.17^b^	6.97 ± 0.30^a^	5.62 ± 0.29^c^	ngd	ngd	ngd
CTL447	ngd	2.6 0± 0.08^e^	4.64 ± 0.15^d^	6.51 ± 0.13^c^	8.98 ± 0.15^a^	7.29 ± 0.02^b^	ngd	ngd
DQC528	ngd	3.94 ± 0.14^e^	7.03 ± 0.22^c^	7.81 ± 0.04^b^	11.14 ± 0.17^a^	4.34 ± 0.05^d^	ngd	ngd
DSC750	ngd	3.52 ± 0.13^c^	7.07 ± 0.04^b^	10.96 ± 0.17^a^	11.10 ± 0.12^a^	3.77 ± 0.04^c^	ngd	ngd
FHL295	ngd	0.78 ± 0.02^b^	2.10 ± 0.04^a^	1.91 ± 0.23^a^	0.66 ± 0.04^b^	ngd	ngd	ngd
FVS803	ngd	3.66 ± 0.15^c^	5.39 ± 0.03^b^	6.00 ± 0.14^a^	1.35 ± 0.11^d^	ngd	ngd	ngd
FPL302	ngd	2.34 ± 0.02^d^	5.55 ± 0.02^c^	7.61 ± 0.18^b^	7.94 ± 0.1^a^	ngd	ngd	ngd
GAL401	ngd	0.83 ± 0.15^d^	2.68 ± 0.35^c^	6.19 ± 0.25^b^	8.76 ± 0.23^a^	ngd	ngd	ngd
GCL642	ngd	3.39 ± 0.09^d^	5.95 ± 0.14^b^	7.28 ± 0.20^a^	4.21 ± 0.21^c^	ngd	ngd	ngd
GCL448	ngd	1.89 ± 0.17^d^	5.18 ± 0.15^c^	8.57 ± 0.18^a^	6.93 ± 0.10^b^	ngd	ngd	ngd
GPL336	ngd	1.12 ± 0.12^c^	2.64 ± 0.01^b^	4.21 ± 0.07^a^	0.45 ± 0.05^d^	ngd	ngd	ngd
GRL334	ngd	2.96 ± 0.07^c^	6.26 ± 0.10^b^	8.41 ± 0.32^a^	7.99 ± 0.14^a^	ngd	ngd	ngd
GRL403	ngd	2.43 ± 0.13^d^	6.43 ± 0.06^c^	8.96 ± 0.12^a^	8.29 ± 0.17^b^	ngd	ngd	ngd
GRC604	ngd	1.49 ± 0.11^c^	3.48 ± 0.11^b^	5.59 ± 0.02^a^	5.53 ± 0.07^a^	ngd	ngd	ngd
GRS805	ngd	0.41 ± 0.03^b^	3.28 ± 0.22^a^	3.63 ± 0.45^a^	ngd	ngd	ngd	ngd
HCC522	ngd	8.93 ± 0.03^e^	17.36 ± 0.77^d^	24.61 ± 0.47^c^	32.63 ± 0.58^a^	32.41 ± 0.47^a^	28.63 ± 0.12^b^	
HEL801	ngd	6.89 ± 0.16^c^	9.69 ± 0.18^b^	13.17 ± 0.07^a^	12.25 ± 0.78^a^	ngd	ngd	ngd
HEL802	ngd	0.10 ± 0.05^b^	0.43 ± 0.06^b^	1.22 ± 0.27^a^	1.46 ± 0.11^a^	ngd	ngd	ngd
HAL340	ngd	2.79 ± 0.13^c^	6.46 ± 0.20^b^	8.29 ± 0.07^a^	8.48 ± 0.08^a^	ngd	ngd	
HUL417	ngd	1.03 ± 0.13^c^	2.21 ± 0.04^a^	1.49 ± 0.31^b^	0.08 ± 0.03^d^	ngd	ngd	ngd
ITL314	ngd	0.46 ± 0.00^d^	2.92 ± 0.18^c^	4.44 ± 0.96^b^	4.96 ± 0.08^a^	3.05 ± 0.08^c^	ngd	ngd
ILC238	ngd	4.20 ± 0.06^d^	7.65 ± 0.24^c^	10.83 ± 0.24^b^	12.19 ± 0.27^a^	2.51 ± 0.10^e^	ngd	ngd
LSL331	ngd	3.78 ± 0.23^c^	5.79 ± 0.05^b^	7.92 ± 0.17^a^	7.84 ± 0.24^a^	ngd	ngd	ngd
LSL332	ngd	1.17 ± 0.24^d^	3.53 ± 0.16^c^	5.73 ± 0.17^b^	7.84 ± 0.28^a^	ngd	ngd	ngd
LES812	ngd	1.97 ± 0.16^b^	3.07 ± 0.16^a^	2.80 ± 0.09^a^	0.44 ± 0.05^c^	ngd	ngd	ngd
NLL317	ngd	1.05 ± 0.09^c^	3.03 ± 0.07^b^	4.35 ± 0.17^a^	4.17 ± 0.14^a^	ngd	ngd	ngd
OIL347	ngd	1.60 ± 0.06^c^	3.55 ± 0.08^a^	2.85 ± 0.13^b^	1.49 ± 0.09^c^	ngd	ngd	
PFL346	ngd	1.37 ± 0.10^d^	3.08 ± 0.04^c^	4.95 ± 0.13^a^	3.67 ± 0.08^b^	ngd	ngd	
PHL322	ngd	8.18 ± 0.23^d^	21.36 ± 0.24^c^	29.28 ± 0.20^b^	36.64 ± 0.91^a^	35.94 ± 0.66^a^	35.61 ± 0.20^a^	ngd
PHC571	ngd	9.89 ± 0.12^f^	22.98 ± 0.16^e^	30.46 ± 0.90^c^	34.23 ± 0.30^b^	40.30 ± 0.09^a^	26.21 ± 0.62^d^	ngd
PNS806	ngd	2.69 ± 0.22^c^	4.39 ± 0.29^a^	3.35 ± 0.20^b^	0.26 ± 0.09^d^	ngd	ngd	ngd
PAC891	0.22 ± 0.04^b^	0.34 ± 0.01^b^	1.17 ± 0.27^a^	1.47 ± 0.31^a^	0.38 ± 0.29^b^	ngd	ngd	ngd
PAC854	0.10 ± 0.02^b^	0.26 ± 0.11^b^	1.02 ± 0.23^a^	0.69 ± 0.13^a^	0.09 ± 0.05^b^	ngd	ngd	ngd
PCC884	0.47 ± 0.01^c^	1.58 ± 0.07^b^	2.62 ± 0.59^ab^	3.72 ± 0.95^a^	3.50 ± 0.37^a^	ngd	ngd	ngd
PCL660	0.85 ± 0.03^c^	1.05 ± 0.02^c^	3.34 ± 0.30^b^	5.07 ± 0.27^a^	3.59 ± 0.10^b^	ngd	ngd	ngd
PCC897	ngd	0.24 ± 0.08^c^	1.54 ± 0.45^b^	2.08 ± 0.25^b^	2.85 ± 0.10^a^	0.58 ± 0.07^c^	ngd	ngd
PDC855	0.40 ± 0.06^c^	0.90 ± 0.08^c^	4.17 ± 0.22^b^	7.09 ± 0.60^a^	6.33 ± 0.29^a^	ngd	ngd	ngd
PEC847	0.22 ± 0.02^c^	0.57 ± 0.10^c^	2.72 ± 0.33^b^	4.67 ± 0.18^a^	2.85 ± 0.01^b^	ngd	ngd	ngd
PEL063	ngd	0.38 ± 0.05^d^	1.60 ± 0.00^c^	2.96 ± 0.31^a^	2.51 ± 0.20^b^	ngd	ngd	ngd
PEC810	0.29 ± 0.04^c^	0.70 ± 0.07^c^	2.10 ± 0.10^b^	3.15 ± 0.37^a^	2.31 ± 0.14^b^	ngd	ngd	ngd
PEC856	0.60 ± 0.04^c^	1.28 ± 0.14^b^	4.16 ± 0.43^a^	4.54 ± 0.12^a^	0.41 ± 0.11^c^	ngd	ngd	ngd
PFC860	0.56 ± 0.06^b^	1.02 ± 0.11^ab^	1.32 ± 0.08^a^	1.15 ± 0.38^a^	0.09 ± 0.00^c^	ngd	ngd	ngd
PNL126	0.21 ± 0.06^c^	0.45 ± 0.04^c^	1.39 ± 0.24^a^	1.04 ± 0.11^b^	0.13 ± 0.02^c^	ngd	ngd	ngd
PNC816	0.22 ± 0.03^bc^	0.56 ± 0.08^b^	1.12 ± 0.42^a^	0.46 ± 0.06^b^	0.03 ± 0.02^c^	ngd	ngd	ngd
POC843	0.77 ± 0.31^c^	2.33 ± 0.13^bc^	4.42 ± 0.38^ab^	5.89 ± 0.20^a^	4.33 ± 0.06^ab^	ngd	ngd	ngd
POC847	0.68 ± 0.10^cd^	1.46 ± 0.24^bc^	6.43 ± 0.19^a^	7.28 ± 0.16^a^	1.80 ± 0.74^b^	0.26 ± 0.00^d^	ngd	ngd
POL067	1.33 ± 0.25^d^	2.64 ± 0.05^c^	7.26 ± 0.08^a^	7.36 ± 0.43^a^	4.00 ± 0.63^b^	ngd	ngd	ngd
PPL111	0.66 ± 0.08^d^	1.06 ± 0.35^d^	4.09 ± 0.25^c^	7.01 ± 0.15^a^	6.24 ± 0.26^b^	ngd	ngd	ngd
PPC823	0.70 ± 0.05^b^	0.91 ± 0.23^b^	3.37 ± 0.40^a^	3.36 ± 0.82^a^	0.94 ± 0.09^b^	ngd	ngd	ngd
PSC757	0.64 ± 0.22^bc^	1.07 ± 0.36^ab^	1.35 ± 0.08^a^	1.18 ± 0.14^a^	0.43 ± 0.01^cd^	ngd	ngd	ngd
PTC822	0.36 ± 0.07^c^	0.54 ± 0.10^c^	1.54 ± 0.47^b^	3.35 ± 0.50^a^	2.01 ± 0.02^b^	ngd	ngd	ngd
SHC608	ngd	7.73 ± 0.15^c^	12.28 ± 0.16^a^	12.29 ± 0.11^a^	8.37 ± 0.24^b^	0.21 ± 0.00^d^	ngd	ngd
THC610	ngd	3.76 ± 0.23^e^	6.90 ± 0.10^c^	8.47 ± 0.07^b^	9.66 ± 0.42^a^	5.44 ± 0.11^d^	ngd	ngd
TLL473	ngd	2.17 ± 0.06^e^	4.55 ± 0.05^d^	6.55 ± 0.10^c^	8.01 ± 0.16^a^	7.47 ± 0.05^b^	ngd	ngd
TVL469	ngd	4.60 ± 0.09^d^	6.89 ± 0.14^c^	8.49 ± 0.14^b^	8.88 ± 0.13^a^	0.38 ± 0.10^e^	ngd	ngd
TVC614	ngd	5.39 ± 0.49^c^	10.53 ± 0.18^a^	11.24 ± 0.23^a^	10.93 ± 0.58^a^	1.52 ± 0.16^b^	ngd	ngd
TLC616	ngd	5.55 ± 0.17^d^	8.01 ± 0.12^c^	9.29 ± 0.06^b^	10.25 ± 0.12^a^	0.48 ± 0.02^e^	ngd	ngd

**Table 3:** Mycelium linear growth rates (*K*_*r*_, mm day^−1^) for 60 strains of macrofungi growing on PDA and on OMW-based substrates prepared in various dilutions in water (v/v); ngd: no growth detected. Values are expressed as means ± standard deviation of means, *n* = 3. Lack of common superscripts indicates statistically significant differences (Gabriel's *t*-test, *P* < 0.05) for comparisons of treatment means between different strains (numbers) and different substrates (lowercase letters).

Strain	Substrate			
	PDA	25% OMW	50% OMW	100% OMW
ABC521	10.56 ± 0.09^5,a^	6.31 ± 0.15^6,7,8,b^	4.59 ± 0.08^6,7,8c^	2.64 ± 0.05^3,4,d^
ABL436	11.86 ± 0.17^4,a^	9.03 ± 0.10^4,5,b^	7.65 ± 0.28^1c^	5.15 ± 0.05^1,d^
ACL834	3.81 ± 0.38^16,17,18,19,a^	2.38 ± 0.13^15,16,b^	1.81 ± 0.19^13,14,b^	ngd
AML472	4.88 ± 1.00^13,14,15,16,a^	ngd	ngd	ngd
CTL447	9.20 ± 0.16^6a^	6.84 ± 0.06^6,b^	4.16 ± 0.10^7,8,9c^	2.40 ± 0.13^4,5,d^
DQC528	10.96 ± 0.10^5,a^	6.04 ± 0.06^7,8,9,b^	5.94 ± 0.08^3,4b^	5.23 ± 0.18^1c^
DSC750	11.19 ± 0.46^4,5,a^	2.87 ± 0.04^14,15,b^	ngd	ngd
FHL295	1.88 ± 0.12^24,25,26,27,a^	ngd	ngd	ngd
FVS803	5.71 ± 0.34^11,12,13,a^	3.98 ± 0.19^12,13,b^	2.88 ± 0.26^11c^	ngd
FPL302	8.04 ± 0.14^7,8,9,a^	0.40 ± 0.00^22,23,24,b^	ngd	ngd
GAL401	9.03 ± 0.20^6,7,a^	6.05 ± 0.29^6,7,8,9,b^	3.88 ± 0.18^9,c^	2.51 ± 0.12^4,d^
GCL642	6.20 ± 0.19^11,12,a^	2.44 ± 0.33^15,16,b^	1.70 ± 0.13^13,14,c^	ngd
GCL448	7.52 ± 0.18^9,10,a^	6.62 ± 0.17^6,7,a^	5.45 ± 0.62^4,5b^	2.29 ± 0.09^4,5c^
GPL336	2.57 ± 0.23^22,23,24,25,a^	1.68 ± 0.06^16,17,18b^	0.90 ± 0.03^16,17,18,19c^	0.54 ± 0.06^7,8,9,10,d^
GRL344	7.73 ± 0.42^8,9,a^	5.43 ± 0.91^9,10,b^	4.83 ± 0.33^5,6,b^	2.32 ± 0.49^4,5,c^
GRL403	8.12 ± 0.03^7,8,9,a^	5.87 ± 0.00^7,8,9,b^	3.96 ± 0.06^8,9,c^	2.14 ± 0.14^4,5,d^
GRC604	4.62 ± 0.10^14,15,16,17,a^	3.57 ± 0.08^13,14,b^	2.59 ± 0.18^11,12c^	0.96 ± 0.15^6,7d^
GFS805	3.24 ± 0.19^17,18,19,20,a^	ngd	ngd	ngd
HCC522	32.99 ± 0.57^1,a^	12.12 ± 0.10^1,b^	1.05 ± 0.00^15,16,17,18c^	ngd
HEL801	13.96 ± 0.07^2,a^	8.27 ± 0.89^5,b^	5.51 ± 0.21^4c^	3.16 ± 0.12^2,3d^
HEL802	1.50 ± 0.28^25,26,27,28,a^	ngd	ngd	ngd
HAL340	7.58 ± 0.04^8,9,10,a^	3.62 ± 0.13^13,14,b^	2.08 ± 0.09^12,13c^	0.98 ± 0.03^6,7d^
HUL417	1.87 ± 0.02^24,25,26,27,a^	ngd	ngd	ngd
ITL314	4.45 ± 0.04^14,15,16,17,a^	0.08 ± 0.03^24,b^	ngd	ngd
ILC238	13.42 ± 0.50^2,3,a^	10.04 ± 0.05^2,3,b^	6.80 ± 0.22^2,c^	0.70 ± 0.00^7,8,9,d^
LSL331	4.19 ± 0.79^15,16,17,18,a^	ngd	ngd	ngd
LSL332	6.53 ± 0.48^10,11,a^	2.86 ± 0.14^14,15,b^	2.71 ± 0.08^11,12,b,c^	1.96 ± 0.33^5,c^
LES812	2.33 ± 0.20^23,24,25,26,a^	ngd	ngd	ngd
NLL317	3.95 ± 0.22^16,17,18,19a^	1.70 ± 0.34^16,17,18b^	ngd	ngd
OIL347	2.25 ± 0.05^23,24,25,26,a^	1.49 ± 0.04^17,18,19,b^	0.80 ± 0.00^16,17,18,19,c^	0.42 ± 0.08^8,9,10,d^
PFL346	3.22 ± 0.05^20,21,22,23,a^	2.09 ± 0.04^15,16,17,b^	1.35 ± 0.03^14,15,16,c^	0.55 ± 0.03^7,8,9,10,d^
PHL322	36.64 ± 0.92^1,a^	12.57 ± 0.18^1,b^	6.48 ± 0.03^2,3c^	ngd
PHC571	34.23 ± 0.30^1a^	10.83 ± 0.10^2,b^	4.18 ± 0.15^6,7,8,9,c^	ngd
PNS806	3.97 ± 0.38^16,17,18,19,a^	ngd	ngd	ngd
PAC891	2.74 ± 0.11^21,22,23,24,a^	0.44 ± 0.16^22,23,24,b^	ngd	ngd
PAC854	1.02 ± 0.12^a^	ngd	ngd	ngd
PCC884	4.69 ± 0.05^13,14,15,16,a^	2.56 ± 0.32^15,b^	2.06 ± 0.17^14,15,16,17,b^	0.23 ± 0.05^9,10,c^
PCL660	2.55 ± 0.68^22,23,24,25,a^	0.06 ± 0.01^22,23,24,b^	ngd	ngd
PCC897	2.30 ± 0.23^23,24,25,26,a^	1.05 ± 0.12^18,19,20,21b^	0.58 ± 0.16^17,18,19,20c^	ngd
PDC855	5.38 ± 0.28^12,13,14,a^	4.19 ± 0.29^11,12,13,b^	3.09 ± 0.27^10,11,c^	0.78 ± 0.24^6,7,8,d^
PEC847	3.98 ± 0.38^16,17,18,19,a^	0.87 ± 0.06^20,21,22,23,b^	0.32 ± 0.03^19,20,21,c^	ngd
PEL063	3.59 ± 0.24^18,19,20,21,a^	0.90 ± 0.09^19,20,21,22,b^	0.20 ± 0,00^20,21,c^	ngd
PEC810	3.76 ± 0.12^17,18,19,20,a^	1.30 ± 0,34^17,18,19,20,b^	0.42 ± 0.13^18,19,20,c^	ngd
PEC856	1.48 ± 0.04^26,27,28,a^	0.42 ± 0.20^22,23,24,b^	ngd	ngd
PFC860	1.03 ± 0.28^27,28,a^	0.51 ± 0.02^21,22,23,24,b^	0.32 ± 0.03^19,20,21,b^	ngd
PNL126	0.94 ± 0.01^27,28,a^	0.36 ± 0.02^23,24,b^	0.07 ± 0.04^21,c^	ngd
PNC816	0.64 ± 0.04^28,a^	0.15 ± 0.10^22,23,24,b^	ngd	ngd
POC843	3.45 ± 0.69^19,20,21,22,a^	1.74 ± 0.06^16,17,18,b^	0.47 ± 0.41^18,19,20,c^	ngd
POC847	5.10 ± 0.03^12,13,14,a^	1.76 ± 0.07^16,17,18,b^	1.15 ± 0.05^18,19,20,21,c^	ngd
POL067	6.64 ± 0.22^10,11,a^	2.39 ± 0.05^15,16,b^	0.75 ± 0.48^16,17,18,19,c^	0.07 ± 0.03^10,c^
PPL111	7.80 ± 0.21^8,9,a^	2.74 ± 0.05^15,b^	1.67 ± 0.16^13,14,15,c^	ngd
PPC823	3.71 ± 0.28^17,18,19,20,a^	1.56 ± 0.10^17,18,19b^	1.06 ± 0.09^15,16,17,18,c^	0.20 ± 0.00^9,10,d^
PSC757	0.80 ± 0.06^27,28,a^	0.50 ± 0.06^22,23,24,b^	0.30 ± 0.00^19,20,21,c^	ngd
PTC822	2.70 ± 0.24^21,22,23,24,a^	0.97 ± 0.12^18,19,20,21b^	ngd	ngd
SHC608	12.83 ± 0.14^3,a^	5.81 ± 0.07^8,9,b^	4.71 ± 0.03^6,7,c^	3.61 ± 0.08^2,d^
THC610	9.08 ± 0.06^6,7,a^	5.78 ± 0.05^8,9,b^	3.74 ± 0.19^9,10,c^	2.62 ± 0.02^4,d^
TLL473	7.40 ± 0.12^9,10,a^	4.93 ± 0.03^10,11,b^	4.80 ± 0.00^10,11,b^	1.24 ± 0.10^6,c^
TVL469	8.64 ± 0.18^6,7,8,a^	5.61 ± 0.03^8,9,10,b^	4.37 ± 0.03^6,7,8,9,c^	3.61 ± 0.08^2,d^
TVC614	8.98 ± 0.18^6,7,a^	4.42 ± 0.04^11,12,b^	4.14 ± 0.05^7,8,9,b^	3.37 ± 0.07^2,c^
TLC616	13.41 ± 0.20^2,3,a^	9.34 ± 0.10^3,4,b^	6.77 ± 0.07^2,c^	2.61 ± 0.06^4,d^

**Table 4:** Biomass production by eight selected strains of macrofungi in OMW (25% v/v) substrates and pH, electric conductivity (E.C.), plant-seed germination index (G.I.), and biodegradation-related aspects as measured in five different time periods during fungal growth. *T*_1_ to *T*_5_ correspond to 4, 8, 12, 16, and 20 days for HCC522 and PHL322, to 5, 10, 15, 20, and 25 days for ABL436, GCL448, HEL801, and ILC238, and to 6, 12, 18, 24, and 30 days for PDC855 and PPL111. Noninoculated substrates served as controls. N.a.: not applicable. Values are expressed as means ± standard deviation of means, *n* = 4. Lack of common superscripts indicates statistically significant differences (Gabriel's *t*-test, *P* < 0.05) for comparisons of treatment means between different strains (capital letters) and different time periods (lowercase letters).

Parameter	Time	ABL436	GCL448	HCC522	HEL801	ILC238	PHL322	PDC855	PPL111	Control
pH	*T*_1_	5.57 ± 0.06^BCa^	5.55 ± 0.10^BCab^	5.62 ± 0.00^ABa^	5.55 ± 0.01^ABCa^	5.44 ± 0.10^Cb^	5.65 ± 0.01^Aa^	5.64 ± 0.00^Aa^	5.60 ± 0.00^Ba^	5.65 ± 0.00^a^
	*T*_2_	5.49 ± 0.10^Cab^	5.50 ± 0.00^Cab^	5.59 ± 0.00^Ba^	5.53 ± 0.02^Ca^	5.25 ± 0.05^Dbc^	5.65 ± 0.01^Aa^	5.60 ± 0.00^Bb^	5.50 ± 0.00^Cb^	5.65 ± 0.00^a^
	*T*_3_	5.35 ± 0.05^Bbc^	5.43 ± 0.05^ABb^	5.53 ± 005^Ab^	5.52 ± 0.02^Aa^	5.07 ± 0.06^Bc^	5.40 ± 0.00^Bb^	5.50 ± 0.00^Ac^	5.44 ± 0.00^Bc^	5.65 ± 0.01^a^
	*T*_4_	5.29 ± 0.12^BCc^	5.40 ± 0.05^Bb^	5.40 ± 0.00^Bc^	5.52 ± 0.01^Aa^	5.00 ± 0.05^Dc^	5.18 ± 0.01^Cc^	5.50 ± 0.00^Ac^	5.18 ± 0.01^Cd^	5.65 ± 0.00^a^
	*T*_5_	5.20 ± 0.05^Cc^	5.30 ± 0.05^Bb^	5.13 ± 0.12^Cd^	5.50 ± 0.02^Aa^	4.48 ± 0.06^Dc^	5.00 ± 0.00^CDd^	5.48 ± 0.01^Ac^	5.14 ± 0.00^Cd^	5.64 ± 0.01^a^

E.C. (mS cm^−1^)	*T*_1_	4.25 ± 0.03^Ae^	3.80 ± 0.10^ABe^	2.63 ± 0.01^Ce^	4.09 ± 0.04^Ad^	2.52 ± 0.04^Ce^	2.60 ± 0.03^Ce^	2.21 ± 0.01^Ee^	2.45 ± 0.06^CDd^	1.80 ± 0.00^b^
	*T*_2_	4.47 ± 0.02^Ad^	4.09 ± 0.03^Cd^	3.27 ± 0.01^Bd^	4.21 ± 0.03^Bd^	3.05 ± 0.02^Fd^	3.03 ± 0.05^Fd^	2.75 ± 0.03^Gd^	2.89 ± 0.02^Gc^	1.82 ± 0.00^b^
	*T*_3_	4.92 ± 0.04^Ac^	4.31 ± 0.07^Bc^	4.05 ± 0.03^Cc^	4.40 ± 0.04^Bc^	4.17 ± 0.02^Cc^	3.68 ± 0.05^Ec^	3.02 ± 0.04^Fc^	3.45 ± 0.10^Fb^	1.89 ± 0.02^a^
	*T*_4_	5.27 ± 0.02^Ab^	4.53 ± 0.09^BCb^	4.87 ± 0.01^Bb^	4.62 ± 0.05^Cb^	4.84 ± 0.03^Bb^	4.19 ± 0.13^Db^	3.33 ± 0.06^Eb^	3.99 ± 0.0^ab^	1.94 ± 0.03^a^
	*T*_5_	5.97 ± 0.05^Aa^	4.79 ± 0.07^Da^	5.67 ± 0.02^Ba^	4.85 ± 0.12^Da^	5.54 ± 0.01^Ca^	4.97 ± 0.05^Da^	3.85 ± 0.01^Fa^	4.23 ± 0.08^Ea^	1.95 ± 0.03^a^

Biomass (g)	*T*_1_	0.05 ± 0.01^Ac^	0.02 ± 0.00^Ec^	0.03 ± 0.00^BCe^	0.02 ± 0.00^Ec^	0.04 ± 0.00^ABd^	0.03 ± 0.00^CDe^	0.02 ± 0.00^DEc^	0.02 ± 0.00^DEd^	n.a.
	*T*_2_	0.15 ± 0.02^Ab^	0.03 ± 0.00^Cbc^	0.05 ± 0.00^Cd^	0.04 ± 0.01^BCb^	0.11 ± 0.01^Bc^	0.04 ± 0.00^Cd^	0.04 ± 0.00^BCb^	0.03 ± 0.01^Ccd^	n.a.
	*T*_3_	0.2 ± 0.02^Aa^	0.04 ± 0.01^Fb^	0.09 ± 0.00^Dc^	0.06 ± 0.02^EFb^	0.14 ± 0.01^Bb^	0.07 ± 0.00^Ec^	0.11 ± 0.01^Ca^	0.04 ± 0.00^Fc^	n.a.
	*T*_4_	0.21 ± 0.01^Aa^	0.12 ± 0.01^BCa^	0.15 ± 0.00^Bb^	0.13 ± 0.03^BCa^	0.15 ± 0.01^Ba^	0.11 ± 0.00^Cb^	0.14 ± 0.02^Ba^	0.11 ± 0.00^Cb^	n.a.
	*T*_5_	0.21 ± 0.01^Aa^	0.13 ± 0.01^Ca^	0.20 ± 0.01^Aa^	0.15 ± 0.02^BCa^	0.17 ± 0.01^Ba^	0.16 ± 0.01^Ba^	0.20 ± 0.02^ABa^	0.23 ± 0.01^Aa^	n.a.

Decolorization (%)	*T*_1_	−25.82 ± 4.37^Fe^	2.41 ± 0.32^Be^	−0.99 ± 0.02^De^	14.23 ± 1.93^Ab^	−6.59 ± 0.41^Ed^	−2.35 ± 0.39^Ce^	−28.24 ± 1.88^Gd^	−23.22 ± 1.89^Fd^	0.44 ± 0.04^b^
	*T*_2_	48.08 ± 3.71^Ad^	4.57 ± 0.54^Ed^	4.55 ± 1.20^Ed^	18.36 ± 0.84^Bb^	8.52 ± 1.30^Dc^	1.78 ± 0.25^Fd^	13.76 ± 1.52^Cc^	−21.44 ± 3.34^Gd^	0.68 ± 0.11^ab^
	*T*_3_	56.84 ± 0.71^Ac^	7.31 ± 0.87^Fc^	17.99 ± 0.45^Dc^	25.21 ± 2.86^Ca^	35.16 ± 3.24^Bb^	11.72 ± 0.46^Ec^	21.09 ± 2.80^Cb^	17.68 ± 1.45^Dc^	0.89 ± 0.20^a^
	*T*_4_	60.99 ± 0.78^Ab^	10.48 ± 1.53^Eb^	25.73 ± 1.32^Db^	27.09 ± 1.31^Da^	51.51 ± 2.57^Ba^	21.65 ± 1.80^Db^	40.64 ± 1.93^Ca^	23.43 ± 2.26^Db^	1.21 ± 0.14^a^
	*T*_5_	64.01 ± 0.32^Aa^	13.86 ± 1.90^Fa^	33.63 ± 2.40^Da^	29.13 ± 0.57^Ea^	55.91 ± 1.64^Ba^	33.16 ± 2.19^Da^	48.12 ± 2.32^Ca^	38.76 ± 3.14^Da^	1.31 ± 0.13^a^

Total phenols reduction (%)	*T*_1_	54.45 ± 3.98^Ad^	2.80 ± 0.41^Dc^	0.50 ± 0.09^Ee^	13.54 ± 3.73^BCc^	15.28 ± 0.79^BCb^	5.06 ± 0.69^CDe^	17.33 ± 1.23^Bd^	11.19 ± 3.55^BCd^	0.38 ± 0.04^d^
	*T*_2_	72.13 ± 3.91^Ac^	48.57 ± 5.48^Bb^	9.57 ± 0.93^Ed^	18.42 ± 1.60^Db^	16.02 ± 1.60^Db^	8.85 ± 0.93^Ed^	32.14 ± 1.62^Cc^	16.61 ± 1.23^Dd^	0.58 ± 0.15^c^
	*T*_3_	81.80 ± 2.59^Ab^	53.80 ± 3.20^Bab^	22.03 ± 1.23^Dc^	19.32 ± 2.28^Db^	54.45 ± 0.64^BCa^	18.60 ± 1.72^Dc^	43.30 ± 3.21^CCb^	46.94 ± 5.96^BCc^	1.10 ± 0.09^b^
	*T*_4_	86.73 ± 2.11^Ab^	60.66 ± 2.16^Ca^	30.69 ± 0.91^Db^	23.83 ± 1.99^Eb^	56.67 ± 0.61^Ca^	29.43 ± 2.12^Db^	69.15 ± 2.50^Ba^	56.15 ± 3.86^Cb^	1.45 ± 0.37^ab^
	*T*_5_	92.39 ± 1.39^Aa^	61.03 ± 3.21^Ca^	47.48 ± 1.78^Da^	45.63 ± 3.60^DEa^	58.89 ± 0.58^Ca^	41.74 ± 2.57^Ea^	76.01 ± 2.39^Ba^	70.59 ± 3.60^Ba^	2.14 ± 0.42^a^

G.I. (%)	*T*_1_	7.58 ± 1.17^Ad^	0.00	0.78 ± 0.09^Cd^	0.00	6.51 ± 0.68^Ad^	1.42 ± 0.06^Bd^	0.71 ± 008^Cd^	0.73 ± 0.14^ Cc^	0.00
	*T*_2_	9.23 ± 0.74^Ad^	6.62 ± 1.31^Bb^	2.77 ± 0.41^Dcd^	5.26 ± 0.94^Bb^	8.78 ± 1.20^Ad^	3.27 ± 0.16^Cc^	1.49 ± 0.10^Ec^	1.85 ± 0.36^Ec^	0.00
	*T*_3_	20.47 ± 0.57^cA^	8.91 ± 0.87^Cb^	4.05 ± 0.71^Dbc^	6.41 ± 1.16C^Db^	14.49 ± 0.47^Bc^	3.91 ± 0.40^Dc^	2.13 ± 0.41^Ec^	4.27 ± 0.85^Db^	0.00
	*T*_4_	24.89 ± 1.57^Ab^	13.28 ± 0.47^Ba^	5.92 ± 1.02^CDab^	7.33 ± 0.54^Cb^	15.78 ± 0.12^Bb^	6.76 ± 0.16^Cb^	4.75 ± 0.51^Db^	6.76 ± 1.52^Ca^	0.00
	*T*_5_	30.01 ± 0.97^Aa^	17.57 ± 1.47^Ba^	8.11 ± 2.03^Ca^	15.01 ± 2.12^Ba^	17.04 ± 0.78^Ba^	8.89 ± 0.26^Ca^	8.11 ± 0.71^Ca^	8.89 ± 0.94^Ca^	0.00

**Table 5:** Coefficient of determination (*r* ^2^) values in comparison between all degradation parameters (biomass, total phenolics reduction, decolorization, and germination index) and enzyme activities on the basis of the entire dataset obtained from all eight macrofungi examined. *Statistically significant at 5%; **statistically significant at 1%.

OMW degradation parameters and enzymes	Fungal biomass	Laccase activity	Mn independent peroxidase activity	Mn peroxidase activity	Decolorization	Total phenolics reduction	Germination index
Fungal biomass	—	0.50**	0.56**	0.56**	0.86**	0.76**	0.88**
Laccase activity		—	0.00	0.28	0.34*	0.63**	0.51**
Mn independent peroxidase activity			—	0.55**	0.46**	0.32*	0.60**
Mn peroxidase activity				—	0.33*	0.47**	0.62**
Decolorization					—	0.71**	0.71**
Total phenolics reduction						—	0.84**
Germination index							—
